# *Tamaricicola fenicei* sp. nov. (Pleosporaceae, Pleosporales), a New Marine Fungus with Significant Antiviral Activity

**DOI:** 10.3390/jof11110801

**Published:** 2025-11-11

**Authors:** Marcella Pasqualetti, Martina Braconcini, Susanna Gorrasi, Paolo Barghini, Emilia Palazzotto, Donatella Ferraro, Domenico Schillaci

**Affiliations:** 1 Department of Biological and Ecological Sciences, University of Tuscia, Largo dell’Università snc, 01100 Viterbo, Italy; martina.braconcini@unitus.it (M.B.); gorrasi@unitus.it (S.G.); barghini@unitus.it (P.B.); 2 Laboratory of Ecology of Marine Fungi (CoNISMa), University of Tuscia, Largo dell’Università snc, 01100 Viterbo, Italy; 3 Microbiology Section, Department of Health Promotion, Mother and Child Care, Internal Medicine and Medical Specialties ‘G. D’Alessandro’, University of Palermo, 90133 Palermo, Italy; emilia.palazzotto@unipa.it (E.P.); donatella.ferraro@unipa.it (D.F.); 4 Department of Biological, Chemical and Pharmaceutical Sciences and Technologies (STEBICEF), University of Palermo, Via Archirafi 32, 90123 Palermo, Italy; domenico.schillaci@unipa.it

**Keywords:** marine fungi, new species, *Tamaricicola fenicei*, antiviral activity, antibiofilm activity

## Abstract

In this study, seven Pleosporaceae strains isolated from the seagrass *Posidonia oceanica* and the jellyfish *Pelagia noctiluca* in the central Tyrrhenian Sea were characterized using a polyphasic approach (morpho-physiological, molecular, and phylogenetic analyses). Based on multi-locus phylogenetic inference and morphological characters, a new species, *Tamaricicola fenicei* sp. nov. was proposed. Multi-locus phylogenetic analyses, using the nuclear ribosomal regions of DNA (nrITS1-nr5.8S-nrITS2, nrLSU, and nrSSU) as well as the *rpb2* and *tef-1α* gene sequences, strongly supported the new taxon. The phylogenetic inference, estimated using Maximum Likelihood and Bayesian Inference, clearly indicates that *Tamaricicola fenicei* sp. nov. forms a distinct clade within the monospecific genus *Tamaricicola*. The antimicrobial activity of the chloroformic and butanolic extracts from malt agar cultures of the new species exhibited interesting antiviral and antibiofilm properties. In particular, a MIC of 3.0 µg/mL was observed against the Echovirus E11 in Vero-76 cells; moreover, a biofilm BIC_50_ reduction at 53 µg/mL was observed against *Staphylococcus aureus* ATCC 25923.

## 1. Introduction

Marine environments represent a huge reservoir of biological and biochemical diversity, which is yet mostly unexplored. Occupying about 70% of the surface of our planet, the oceans support a substantial portion of Earth’s biodiversity. To date, 245,000 species have been reported [1]. Several researchers have recently emphasized the need to expand our knowledge of marine fungal biodiversity, which is estimated to exceed 12,500 species, although only about 2000 of them have been formally recorded to date [2,3]. Understanding the biodiversity and species distribution of marine fungi is crucial for gaining deeper insights into their ecological roles. The vast and largely unexplored diversity of marine fungi, shaped by unique environmental conditions, offers a rich source of novel enzymes and bioactive metabolites with promising biotechnological and pharmaceutical applications, including the development of new drugs against multi-drug-resistant infections [4,5,6,7,8,9,10,11].

The majority of known marine fungi belongs to the phylum Ascomycota (82%), followed by Basidiomycota (8.3%) and Microsporidia (6.7%); while phyla such as Chytridiomycota and Mucoromycota are less common, each representing less than 1.5% of recorded species [12]. The class Dothideomycetes (Ascomycota) includes numerous marine species, particularly in the orders Pleosporales and Dothideales. The family Pleosporaceae is one of the largest within the order Pleosporales, comprising 23 genera and more than 200 species [13]. The members of the family are morphologically characterized by the presence of globose ascomata with thick walled peridium, bitunicate cylindrical asci producing eight, septate, sometimes muriforms, ascospores [14,15]. For a long time, the genera of the family were primarily distinguished by their ascospore features. However, phylogenetic molecular investigations have recently led to include in the Pleosporaceae family new taxa that produce only asexual structures, such as the two novel genera *Neostemphylium* and *Scleromyces*, isolated from freshwater sediments in Spain [16]. The species of Pleosporaceae generally exhibit dematiaceous hyphomycetous anamorphs that produce phragmo- or dyctioconidia from blastic conidiogenous cells on macronematous conidiophores, even if coelomycetous anamorphs with phialidic or anellydic conidia have also been reported [15].

The pleosporalen species exhibit a wide range of behaviors; they can be saprophytic, endo-/epiphytic, or parasitic on a variety of hosts in both terrestrial and marine environments. The family includes common ubiquitous species distributed worldwide (e.g., *Alternaria* spp.) and others with a narrower distribution such as the species of the genus *Tamaricicola* [15,17].

The genus *Tamaricicola* was introduced to accommodate a new taxon *T. muriformis* isolated from *Tamarix gallica* [17]. Up to this date the genus is monospecific even if some new strains, possibly representing new lineages, have been isolated from *Limonium majus* and *L. insigne* (CF-288959, CF-288916, CF-090279), from lungs of *Antigone canadensis tabida* (Greater sandhill crane) and from the ascidian *Ciona intestinalis* (CHG59) [18,19,20]. Some strains of *Tamaricicola* (CF-288959 and CHG59) have been tested for their biological activity; in particular, antimicrobial and anticancer activities were tested. It is worth noting that *Tamaricicola* sp. strain CF-288959 showed interesting antifungal activity against *Candida albicans* [18], while *Tamaricicola* sp. strain CHG59 exhibited antibacterial activity against methicillin-resistant *Staphylococcus aureus* and *Acinetobacter baumannii* [19]. To the best of our knowledge, no antiviral screening was carried out using *Tamaricicola* extracts.

The search for new antiviral compounds is of particular importance due to the clinical relevance of several infections linked to the emergence of new variants and the lack of effective therapies. Enteroviruses belonging to the Picornaviridae family are well-recognized for their capacity to cause a wide spectrum of human diseases. Due to their high variability, no universal vaccines exist for non-polio enteroviruses. Therefore, there is an urgent need to identify new, effective compounds with anti-enterovirus activity. Despite extensive research efforts, many compounds, showing promising in vitro anti-Enterovirus activity, have failed to demonstrate comparable efficacy in vivo [21,22]. Echovirus 11 (E11), an enterovirus B species, historically linked to mild febrile illness, has gained renewed attention due to its association with severe neonatal disease. Between 2023 and 2025, ECDC and WHO identified Echovirus 11 as a re-emerging pathogen responsible for severe neonatal infections characterized by fulminant hepatitis and multiorgan failure [23]. Recent studies suggested the global dissemination of a new, highly virulent lineage [24,25,26].

During an extensive investigation aimed at expanding our knowledge of marine fungal biodiversity in the Tyrrhenian Sea, seven strains belonging to the genus *Tamaricicola* have been isolated from the seagrass *Posidonia oceanica* (IG108, IG114) and the jellyfish *Pelagia noctiluca* (PN23, PN28, PN32, PN38, PN39) [3,27]. Preliminary analyses suggested that these strains could represent a novel lineage within the genus [3,27]. This study aimed to characterize this novel species based on morphological, physiological, and molecular analyses. In addition, considering the biotechnological potential of pleosporalen marine fungi [28,29,30,31], the antibacterial, antifungal, and antiviral activities of different extracts were assessed. For this purpose, reference fungal and bacterial strains (*Staphylococcus aureus* ATCC 25923, *Pseudomonas aeruginosa* ATCC 15442, *Escherichia coli* ATCC 25922, *Enterococcus faecalis* ATCC 29212 and the yeast *Candida albicans* ATCC 10231) and a member of the Enterovirus genus were used as test strains.

## 2. Materials and Methods

### 2.1. Fungal Isolates

The fungal strains analyzed in this study were isolated from different substrata collected from the “Cala Cupa” cove (42°22008.0300 N, 10°55004.0900 E), Giglio Island (Tuscan Archipelago, North Tyrrhenian Sea), at 10–15 m depth by scuba divers. Strains IG108 and IG114 were isolated from the leaves of the seagrass *P. oceanica* (15 m depth—May 2015), while strains PN23, PN28, PN32, PN38, and PN39 were isolated from the inner tissues of the jellyfish *P. noctiluca* (10 m depth—May 2019) [3,27]. All strains were cryogenically maintained at −40 °C in the culture collection of microorganisms of the “Laboratory of Ecology of Marine Fungi” (Department of Ecological and Biological Sciences—DEB, University of Tuscia). Strain PN38 (MUT6838) is also preserved in the Mycotheca Universitatis Taurinensis (MUT) culture collection, Italy.

### 2.2. Morphological Analysis on Different Media

Strains had been revitalized and sub-cultured on Potato Dextrose Agar seawater (PDAs; 39 g PDA dissolved in 1 L of filtered seawater) at 23 °C. Morphological analyses were carried out on PDAs, Malt Extract Agar seawater (MEAs; 30 g malt extract, 5 g peptone, 15 g agar, dissolved in 1 L of seawater), Malt Extract Broth seawater, Corn Meal Agar seawater (CMAs; 17 g CMA dissolved in 1 L of seawater) and Oatmeal Agar seawater (OAs; 30 g oatmeal powder, 20 g agar dissolved in 1 L of seawater). All media and components were purchased from Sigma-Aldrich, St. Louis, MO, USA. Petri dishes (5 cm diameter) were inoculated with an agar plug (2 mm^2^) taken from the margin of 15-days-old PDAs cultures and incubated at 23 °C in sealed plastic boxes. Growth was assessed over a 28-day period, and both macroscopic and microscopic traits were recorded. To induce sexual reproduction, *Mycelia sterilia* were inoculated on natural substrata: barks (*Quercus robur*, *Pinus halepensis*), pine needles (*P. halepensis*), twigs (*Tamarix gallica*, *T. africana*) and *Posidonia* leaves [32]. All substrata were cut into small pieces (3 × 1 cm), sterilized, and placed on the surface of well-developed colonies. The plates were incubated for 1 month at 23 °C to allow substrata colonization. Following this period, some of the inoculated fragments were transferred into tubes containing 20 mL of sterile seawater to simulate natural conditions, while others were transferred into moist chambers and further incubated for 18 months at 23 °C. All inoculated fragments were checked regularly. All experiments were carried out in triplicate. Microscopic characterization of somatic and reproductive structures was carried out on slides with lactic acid. Conidioma sections (10–15 μm) were obtained using Leica cryostat (Leica Biosystems, Nussloch, Germany), reproductive structures were collected, embedded in OCT-Optimal Cutting Temperature-mounting media (VWR, International, Radnor, PA, USA), frozen in liquid N_2_, and maintained at −20 °C before sectioning. All samples were observed using a Zeiss AxioPhot microscope (Carl Zeiss Microscopy GmbH, Jena, Germany), and micrographs were taken with a Jenoptik ProgRes^®^ camera (JenOptik AG, Jena, Germany). Dimensions of structures in species descriptions were based on at least 35 measurements.

### 2.3. Molecular Analysis

Genomic DNA was extracted from fresh mycelium using the ZR Fungal/Bacterial DNA MiniPrep Kit (Zymo Research, Irvine, CA, USA) following the manufacturer’s instructions. The extracted DNA was spectrophotometrically quantified (Qubit, Thermo Fisher Scientific, Waltham, MA, USA) and stored at −20 °C. For each strain several loci were amplified and sequenced: ITSrDNA, LSUrDNA, SSU rDNA, *tef-1α* (translation elongation factor) and *rpb2* (RNA polymerase II subunit). Primers for the amplifications were reported in Table 1.

Amplifications were performed in a 25 µL reaction volume containing: 2 µL of genomic DNA, 0.5 µL of each primer (10 µM), 2.5 µL of MgCl2 (25 mM), 1.5 µL of 5× buffer, 0.5 µL of dNTPs (10 mM), 0.2 µL of Go-Taq Polymerase (Promega, Madison, WI, USA); the final volume was reached by adding ultrapure water. Amplifications were performed using a 2720 Thermal Cycler (Applied Biosystem, Waltham, MA, USA) using different PCR conditions (Table 1).

Amplicons were purified (E.Z.N.A. Cycle Pure kit, Omega Bio-tek, Norcross, GA, USA) and sequenced (Eurofins Genomics, Ebersberg, Germany). The sequences obtained were inspected and trimmed with the Chromas Lite 2.1 program. Newly generated sequences were deposited in GenBank NCBI (National Center for Biotechnology Information) (Table 2).

### 2.4. Sequence Alignment and Phylogenetic Analyses

For the phylogenetic analyses a concatenated dataset of nrSSU, nrITS, nrLSU, *rpb2*, and *tef-1α* sequences (Table 2) including the most representative species of the family Pleosporaceae was used [16,17]. The single gene sequence datasets were aligned with the Clustal X 2.1 software [37]. Alignments were checked and edited with BioEdit Alignment Editor 7.2.5 [38] and manually adjusted in MEGA 11 [39], when necessary. For the multi-locus phylogenetic analysis, alignments of different markers were concatenated with MEGA 11. Phylogenetic inference was estimated using Maximum Likelihood (ML) and Bayesian Inference (BI) as previously reported by Pasqualetti et al. [3]. The best-scoring trees were depicted using FigTree v.1.4 (http://tree.bio.ed.ac.uk/software/figtree/; accessed on 10 August 2025).

### 2.5. Antimicrobial and Antiviral Activity

#### 2.5.1. Cultivation and Extraction

Since no differences in the biological activity among the various *Tamaricicola* strains were recorded, one of them (PN38) was designated as the type strain of the new species and used for the subsequent investigation.

Strain PN38 was cultivated on two different media: MEAs and Glucose Yeast Peptone seawater (GYPs; 1 g glucose, 0.5 g peptone special, 0.1 g yeast extract, 15 g agar dissolved in 1 L of filtered seawater). For each medium fifty plates (9 cm Ø), were inoculated as previously reported, and incubated at 23 °C for 30 and 60 days for MEAs and GYPs, respectively. Subsequently, the fungal cultures were cut into small pieces and extracted overnight with MeOH (1 L × 2 times). The MeOH extract solution was concentrated to dryness by a rotary evaporator. Subsequently the *n*-BuOH, and CHCl_3_ fractions of the extract were obtained.

#### 2.5.2. Antimicrobial Activity Assay

In total, four fractions were tested: *n*-BuOH fraction from MEAs and GYPs and CHCl_3_ fraction from MEAs and GYPs. The minimum inhibitory concentrations (MICs) of extracts were determined using a microdilution method against reference strains-*Staphylococcus aureus* ATCC 25923, *Pseudomonas aeruginosa* ATCC 15442, *Escherichia coli* ATCC 25922, *Enterococcus faecalis* ATCC 29212 and the yeast *Candida albicans* ATCC 10231-as recommended by the Clinical and Laboratory Standards Institute [40]. Each sample was tested at concentrations ranging from 0.3 to 2.5 mg/mL using two-fold serial dilution in Mueller–Hinton broth (Sigma Aldrich) for bacteria and Sabouraud broth (Sigma Aldrich) for yeast [41,42]. The assay was performed in a 96-well plate, using a stock solution of 50 mg/mL of the *n*-BuOH, and CHCl_3_ extract fractions diluted in Dimethyl Sulfoxide (DMSO).

The microbial inoculum was obtained from cultures grown at 37 °C for 24 h on Tryptic Soy Agar (TSA-Sigma Aldrich) for bacteria and Sabouraud agar for the yeast. The concentration was adjusted to 1.5 × 10^8^ CFU/mL (McFarland standard 0.5) using 0.9% NaCl saline solution. Ten microliters of each microbial suspension were used as inoculum. A positive control (microorganism and medium without PN38 extract), a negative control (medium without inoculum), and a substance control (medium with extract without microbial inoculum, to evaluate the absorbance of extracts), were also included in the 96-well plate. Moreover, controls with DMSO (to exclude biological activity of the diluent at the tested concentrations of extract) and with a known antibiotic, Amikacin (for quality control of antibacterial experiments, without comparative purposes) were also made.

The 96-well plates were incubated at 37 °C for 24 h and MICs were determined by a microplate spectrophotometer (GloMax^®^-Multi Detection System, Promega, Madison, WI, USA) as the lowest concentration of extract whose OD, read at 570 nm, was comparable with the negative control wells. Each assay was performed in triplicate. The biofilm inhibition formation was tested on *S. aureus* ATCC 25923. The strain was incubated in Tryptic Soy Broth (TSB) supplemented with 2% (*w*/*v*) glucose at 37 °C for 24 h. After incubation, 2.5 μL of microbial suspension was added to each well of a flat-bottom 96-well plate containing 200 μL of TSB with 2% glucose. Aliquots of each extract at sub-MIC concentration, ranging from 500 to 1.5 μg/mL, were directly added to the wells; positive, negative and substrate control were also included.

The microplates were incubated at 37 °C for 24 h. Following biofilm formation, the wells were washed twice with sterile NaCl 0.9% solution, and the sessile biomass was stained with 100 μL of 0.1% crystal violet solution for 30 min at 37 °C. After incubation, the plate was washed twice, and 200 μL of ethanol was added into each well. The plate was then incubated for 10 min at room temperature, and the optical density (OD) was measured at 540 nm (Glomax Multidetection System TM297). The percentage of biofilm inhibition was determined through the following formula:

$$Inhibition\;\left(\%\right)=\;\frac{OD\;growth\;control\;-\;OD\;sample\;}{OD\;growth\;control}\;\times \;100$$

BIC_50_ (concentration at which the percentage of inhibition of biofilm formation is equal to 50%), was obtained by comparing the ODs of control wells with that of the sample wells at different concentrations, and the value was calculated using AAT Quest Graph^TM^ IC50 Calculator (v.1) (Bioquest, Inc., Sunnyvale, CA, USA) retrieved from https://www.aatbio.com/tools/ic50-calculator (accessed on 1 July 2025). Each assay was performed in triplicate.

#### 2.5.3. Antiviral Activity Assay

For the antiviral assay, as in the antibacterial test, stock solutions (50 mg/mL) of the *n*-BuOH and CHCl_3_ extract fractions of MEAs and GYPs were prepared in dimethyl sulfoxide (DMSO).

**Virus**—Epithelial monkey kidney Vero-76 cells [ATCC CRL 1587, *Cercopithecus aethiops*] were maintained in Dulbecco’s modified Eagle’s medium (DMEM; Sigma-Aldrich) supplemented with 1% penicillin-streptomycin, and 2 mM L-glutamine (Sigma-Aldrich), and 10% fetal bovine serum (FBS; Sigma-Aldrich), at 37 °C and 5% CO_2_. Echovirus 11 lineage 1, was maintained and propagated in Vero-76 cells. The virus was stored in small aliquots at −80 °C. All experimental work involving the virus was conducted with the appropriate biosafety level containment.

**Cytotoxicity evaluation by optical microscopy**—Cytotoxicity of the extracts from different media was assessed by monitoring morphological alterations in Vero-76 cells. In addition, cell viability was determined using the crystal violet staining method. In particular, Vero-76 cells were seeded in a 96-well plate at 2 × 10^4^ cell/mL, in DMEM medium. After 24 h, the cell monolayers were treated with different dilutions (50–0.75 µg/mL) of PN38 extract for 24 h; DMSO was utilized as control. Absorbance was read at 570 nm, and the percentage of cell viability was calculated using the following formula:

$$Cell\;viability\left(\%\right)=\;\frac{Sample\;absorbance\;\left(treated\;samples\right)-Control\;absorbance\;}{Control\;absorbance}\;\times \;100$$

The experiment was conducted in triplicate.

**Cytopathic effect inhibition assay**—The potential antiviral activity of PN38 extract against E11 was evaluated by the cytopathic effect (CPE) inhibition assay in a co-treatment antiviral assay: Vero-76 cells were seeded into 96-well plate (2 × 10^4^ cell/mL) and incubated overnight at 37 °C in a humidified atmosphere containing 5% CO_2_. Different concentrations of PN38 extract (25–0.75 μg/mL) and E11 at a multiplicity of infection (MOI) of 0.01 were added to the cell monolayer and incubated for 48 h at 37 °C in DMEM supplemented with 5% FBS. CPE reduction was evaluated by optical microscopy observation. Also, cells were fixed with 4% formaldehyde for 15 min at room temperature and then stained with 0.1% (*w*/*v*) crystal violet for 30 min at room temperature. The intensity of the crystal violet stain was evaluated by spectrophotometry at 595 nm. The minimal inhibition concentration (MIC) was defined by serial dilution of PN38 extract (25–0.75 μg/mL). Infective events were counted microscopically to evaluate the viability of extract-treated cell cultures infected with E11, as well as untreated cell cultures infected with E11 (Control Virus, CV). Uninfected cell cultures were also used as controls (Control Cells, CC) [43]. All experiments were carried out in triplicate.

**E11 genome quantification in Vero-76 infected cells**—The E11 viral genome copies in the supernatants of infected cells treated with different concentrations of PN38 (25–0.75 μg/mL) were quantified. After 48 h post-infection, viral RNA was extracted from the supernatant of treated and control cells and quantified by qRT-PCR using the automated Elite InGenius one step RNA Enterovirus ELITe MGB^®^ Kit (ELITechGroup, Torino, Italy), according to manufacturer’s protocols. Each experiment was performed in triplicate, and RNA yields are reported as the mean values of three independent assays.

#### 2.5.4. Statistical Analysis

One-way ANOVA and post hoc tests (Tukey) were performed to compare results of assays [44]. Statistical significance was set at *p* < 0.05.

## 3. Results

### 3.1. Phylogenetic Analyses

**Figure 1:**
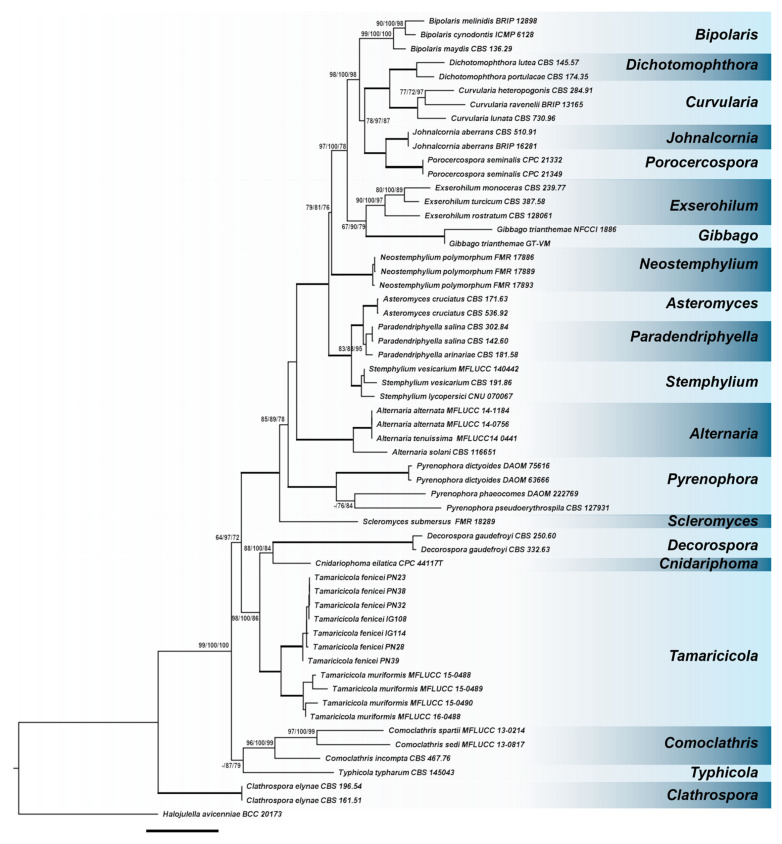
Phylogenetic analysis on nrLSU, nrITS, nrSSU, *rpb2*, and *tef-1α* using the Maximum likelihood method. *Halojulella avicenniae* was used as the outgroup to root the tree. Branch numbers indicate SH-aLRT/BYPP/BS values. Bold branches indicate SH-aLRT/BYPP/BS values of 100/100/100. Bar = expected changes per site (0.06). Evolution models used: part1 TNe + G4; part2 JC + I + G4; part3 TIM2e + G4; part4 TIM2e + I + G4; part5 TNe + I + G4; part6 K2P + I; part7 TNe + G4; part8 TN + F + I + G4.

A preliminary phylogenetic analysis was carried out separately for nrLSU, nrITS, nrSSU, *rpb2*, and *tef-1α*. As no incongruences were observed among the single-locus phylogenetic trees, a multi-locus analysis was performed. The final dataset included 58 strains, representing 37 species, and 21 genera within the family Pleosporaceae. In total, 33 sequences were newly generated, while 176 sequences were retrieved from GenBank (Table 2). The concatenated alignment included 4534 characters (861 for nrLSU, 636 for nrITS, 1020 for nrSSU, 851 for *rpb2*, and 1166 for *tef-1α*) including gaps. Among them, 1337 distinct patterns, 882 parsimony-informative sites, 304 singleton sites, and 3348 constant sites, were observed. ML analysis yielded a best-scoring three with a final optimized likelihood value of −22,563.004. The ML and BI analyses produced generally congruent topologies, thus, only the ML tree with BS and BYPP values was reported (Figure 1).

Phylogenetic analysis of the concatenated dataset showed that the seven isolates under investigation formed a well-supported clade (BS = 100%; BYPP = 100%) within the genus *Tamaricicola* clearly distinguished by *T. muriformis*.

**Figure 2:**
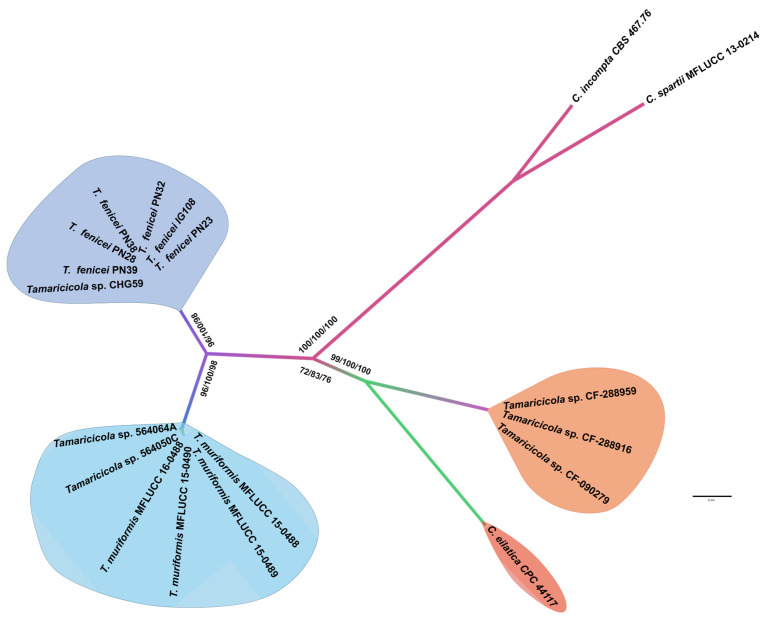
Phylogenetic inference based on nrITS using the Maximum likelihood method. The tree is rooted in the species *Comoclathris incompta* CBS 467.76 and *Comoclathris spartii* MFLUCC 13-0214. Branch numbers represent SH-aLRT/BYPP/BS values. The scale bar indicates the expected number of substitutions per site (0.04).

Although *T. muriformis* remains the only species formally described within the genus to date, several other *Tamaricicola* strains, beyond those investigated in this study, have been reported from marine and terrestrial substrates. Notably, two strains were isolated from avian lungs [20], three from *Limonium* spp. [18], and one from the ascidian *Ciona intestinalis* [19]. These strains were not included in the global phylogenetic analyses carried out for the Pleosporaceae family, as only nrITS sequences are currently available for them. For these reasons, a supplementary ITS-based phylogenetic analysis, incorporating all known *Tamaricicola* strains, was performed (Figure 2). The sixteen strains attributed to the genus were resolved into three distinct and well-supported clades. The strains *Tamaricicola* sp. 56406A and *Tamaricicola* sp. 56405C clustered with *T. muriformis*, while *Tamaricicola* sp. CHG59 grouped with the strains of the newly proposed species *T. fenicei*. The remaining three strains—*Tamaricicola* sp. CF-288959, CF-288916, and CF-090279—appear to represent a separate lineage. Interestingly, this latter lineage shows phylogenetic affinity with the newly established genus and species *Cnidariophoma eilatica*, described from a coral-associated strain [45]. Based on ITS sequence data alone, the three strains (*Tamaricicola* sp. CF-288959, CF-288916, and CF-090279) appear not to belong to *Tamaricicola* but, instead, represent a distinct lineage within or closely related to *Cnidariophoma*.

Phylogenetic analyses strongly indicated that the seven investigated strains belong to a new lineage within the genus *Tamaricicola*: the new species *Tamaricicola fenicei* is herein proposed.

### 3.2. Taxonomy

**Figure 3:**
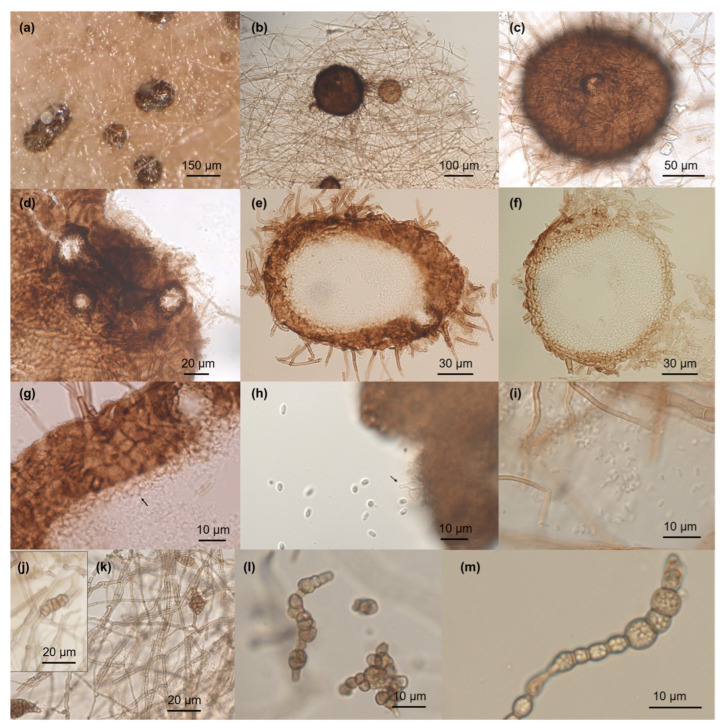
*Tamaricicola fenicei* sp. nov. (**a**)—Conidioma on Corn Meal Agar, white drops of released conidia are present; (**b**)—Conidioma in different stage of maturation; (**c**,**d**)—Conidioma with single and multiple ostiole; (**e**,**f**)—Conidiomata section; (**g**)—Details of conidiomata section; the arrow indicates the conidiogenous cells; (**h**)—Conidiogenous cells (arrow) and conidia; (**i**)—Conidia; (**j**,**k**)—Mycelium and muriform chlamydospores; (**l**,**m**)—Toruloid mycelium with abundant lipids globules.

***Tamaricicola fenicei*** Pasqualetti & Braconcini **sp. nov.** (Figure 3)

**MycoBank.** MB861134

**Etimology.** In honour of the Italian Microbiologist Massimiliano Fenice.

**Type.** Italy, Tuscany, Mediterranean Sea, Giglio Island (Grosseto), Cala Cupa, 4222008.0300 N, 1055004.0900 E, 10 m depth. Isolated from the jellyfish *Pelagia noctiluca*, May 2019, Marcella Pasqualetti. Holotype MUT6838 (strain PN38), living culture permanently preserved in a metabolically inactive state at the Mycotheca Universitatis Taurinenesis (MUT, affiliated to the World Federation Culture Collections WFCC).

**Diagnosis.** *T. fenicei* is introduced to accommodate seven novel strains retrieved on two different substrata in the Tyrrhenian Sea. *T. fenicei* is a biotic marine fungus associated with *P. noctiluca* and *P. oceanica*. Multi-locus phylogenetic analysis showed that *T. fenicei* clustered into a distinct clade in the monospecific genus *Tamaricicola* and differs from its closest phylogenetic neighbour *T. muriformis* by genetic characters in nrITS, nrLSU, nrSSU, *tef-1α*, and *rpb2* sequences and in conidia dimensions as well as in the production of large, thick-walled, and muriform resting spore (chlamydospore). Morphologically, *T. fenicei* resembles the species *T. muriformis* in having similar asexual reproductive structures [17].

**Description.** Growing on *Posidonia oceanica* leaves, internal tissues of *Pelagia noctiluca*, *Quercus robur* and *Pinus halepensis* barks, *P. halepensis* pine needle and twigs of *Tamarix gallica* and *T. africana*.

*Hyphae* 2.2–4.5 µm wide, irregular, septate, sometimes toruloid containing large amount of lipid droplets, sub-hyaline to slightly pigmented. Sexual morph not observed. Asexual morph coelomycetous. *Conidiomata* 80–190 μm diam, pycnidial, superficial or partially immersed, dark brown to black, spheroidal to sub-spheroidal, ostiolate (1–3). *Conidiomatal wall* 10–15 μm wide, comprising few layers of dark brown to hyaline cells of *textura angularis*. *Conidiophores* micronematous, reduced to conidiogenous cells. *Conidiogenous cells* 3–4.5 × 2–4 μm, phialidic, hyaline, smooth, ampulliform. *Conidia* 1.8–4 × 1.5–2.3 μm, ellipsoidal to cylindrical, hyaline, rounded at both ends, 1-celled, smooth-walled, slightly falcate. *Chlamydospores* 18–25 × 7.5–10 μm, muriform, irregular, with several transverse, longitudinal, and oblique septa.

**Figure 4:**
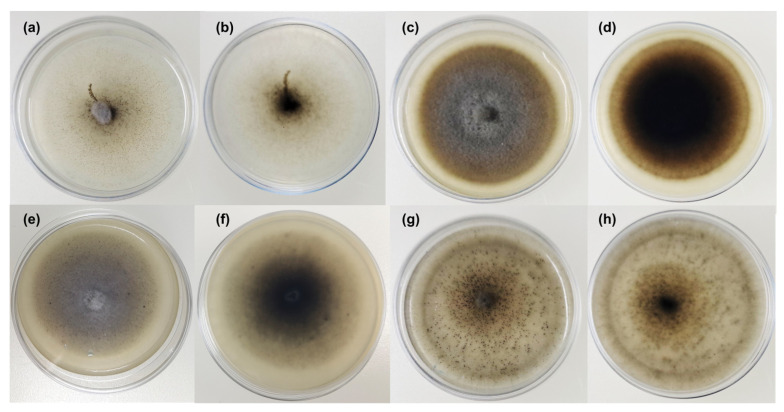
*Tamaricicola fenicei* 21-day-old colonies on different media (Ø 5 cm): (**a**,**b**) CMAs; (**c**,**d**) MEAs; (**e**,**f**) PDAs; (**g**,**h**) OAs. For each substratum front and reverse pictures were reported.

**Colony description** (Figure 4). Colonies on MEAs-PDAs, reaching 48–50 mm diameter after 21 days at 23 °C, plane, umbonate, surface velutinous centrally feltrose, grey, with a light brown marginal area; aerial mycelium sparse, whitish to light brown, mainly in the central area; margins regular moderately deep, reverse dark grey. Soluble pigment and exudates absent. Conidiomata produced on PDAs, not observed on MEAs. Colonies on OAs, reaching 50 mm diameter after 21 days at 23 °C, plane, surface slightly feltrose, whitish, margins regular, reverse white-grey darker in the center. Soluble pigment and exudates absent. Conidiomata produced in very large amounts. Colonies on CMAs, reaching 48 mm diameter after 21 days at 23 °C, surface slightly feltrose, greyish, mycelium prevalently immersed, margins regular, reverse hyaline to greyish. Soluble pigment and exudates absent. Conidiomata present.

**Notes.** No sexual form (ascomata) was detected in artificial and natural substrata. In liquid media the mycelium, prevalently toruloid, shows characteristic rounded cells full of a large amount of small lipidic droplets (Figure 3).

Based on a Megablast search on NCBI nucleotide database, the closest hits of nrITS sequences are Ascomycota sp. Di283-2 (GenBank accession no. OR367423; identities 554/554—100%); Ascomycota sp. San Juan 55-1 (GenBank accession no. KF638538; identities533/533—100%) and *Tamaricicola* sp. strain CHG59 (GenBank accession no. MW064152; identities 494/494—100%).

The closest hits using the nrLSU sequences are Pleosporaceae sp. M306 (GenBank accession no. KJ443126; identities 1276/1306—98%, 7 gaps), *Comoclathris typhicola* MUT<ITA>: 4379 (GenBank accession no. KF636774; identities 1275/1306–98%, 10 gaps) and *Comoclathris typhicola* strain CBS 132.69 (GenBank accession no. JF740325; identities 1275/1306—98%, 10 gaps). The closest hit using the nrSSU sequences is *T. muriformis* isolate IT_9172 (GenBank accession no. KU870908; identities 1043/1049—99%, 6 gaps). The closest hit using the *tef-1α* sequences is *T. muriformis* isolate IT_9175 (GenBank accession no. KU600014; 922/947—97%, 0 gaps). The closest hits using the *rpb2* sequence (ON887328) are *Cnidariophoma eilatica* CPC 44117 (GenBank accession no. OQ627943; identities 610/656—93%, 0 gaps) and *T. muriformis* isolate IT_9173 (GenBank accession no. KU820870; identities 551/579—95%, 0 gaps).

**Additional material examined.** Italy, Tuscany, Mediterranean Sea, Giglio Island (Grosseto), Cala Cupa, 4222008.0300 N, 1055004.0900 E, 10 m depth. Isolated from the jellyfish *P. noctiluca*, May 2019, Marcella Pasqualetti, living culture PN23, PN28, PN32, PN39. Italy, Tuscany, Mediterranean Sea, Giglio Island (Grosseto), Cala Cupa, 4222008.0300 N, 1055004.0900 E, 15 m depth. Isolated from *P. oceanica,* May 2015, Marcella Pasqualetti, living culture IG108, IG114.

### 3.3. Screening for Antimicrobial and Antiviral Activity

The *n*-butanol (*n*-BuOH) and chloroform (CHCl_3_) extracts of strain PN38 cultivated in MEAs and GYPs were evaluated for antimicrobial and antiviral activity. No antiviral, antibacterial or antibiofilm activities were detected at the maximum tested concentrations for the extracts obtained from cultures grown in GYPs medium. Therefore, only the results for extracts derived from MEAs cultures are reported.

To assess antibacterial and antifungal activity the extracts were tested against several pathogenic organisms (*S. aureus* ATCC 25923, *P. aeruginosa* ATCC 15442, *E. coli* ATCC 25922, *E. faecalis* ATCC 29212 and *C. albicans* ATCC 10231) using the microdilution method.

**Figure 5:**
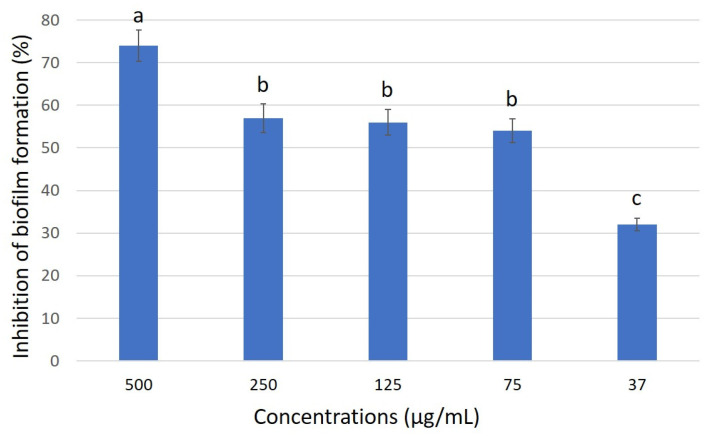
Inhibition of biofilm formation of the strain PN38 (MEAs *n*-BuOH extract) against *S. aureus* ATCC 25923. Different letters represent significant differences (*p* < 0.05, Post Hoc test).

No activity was observed against bacterial and fungal strains at the screening concentration of 2.5 mg/mL for extracts obtained by MEAs cultures. However, an interesting anti-biofilm activity against *S. aureus* ATCC 25923 was observed at sub-MIC concentration of MEAs *n*-BuOH extract with a BIC_50_ value of 53 μg/mL (Figure 5).

**Figure 6:**
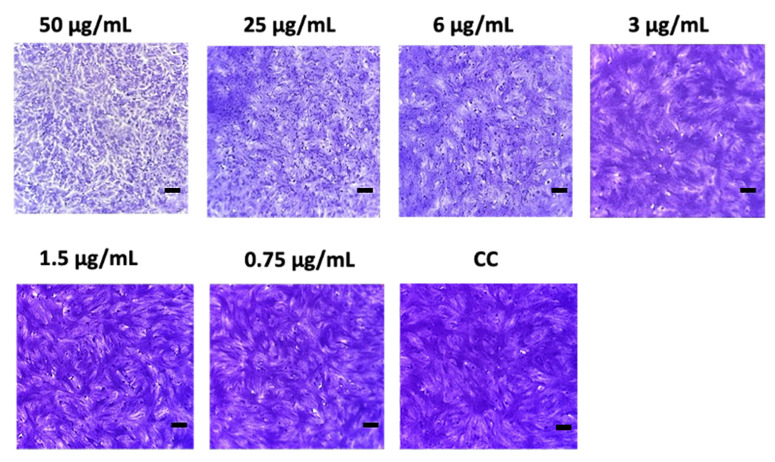
Cytotoxic effects of MEAs CHCl_3_ extract assessed by monitoring morphological alterations in Vero-76 cells. Representative microscopy images of crystal violet stained Vero-76 cells exposed for 24 h to increasing concentrations of PN38 extract, or DMSO (CC) are shown. A strong cytotoxic effect was observed at 50 µg/mL. Scale bar: 100 µm.

The antiviral activity was assessed only on the CHCl_3_ extract due to the cell toxicity of the *n*-BuOH extract. The optical microscopy observation revealed a dose-dependent cytotoxic effect of MEAs CHCl_3_ extract on Vero-76 cells. A marked viability decrease was observed at concentration of 50 µg/mL showing a strong cytotoxic effect (Figure 6). The 25 µg/mL concentration was the first with a reduced cytotoxicity, while 6 μg/mL was the highest non-cytotoxic concentration observed (cell viability > 99%). On the basis of these results, scalar concentrations from 25 to 0.75 µg/mL were used for the antiviral assay.

**Figure 7:**
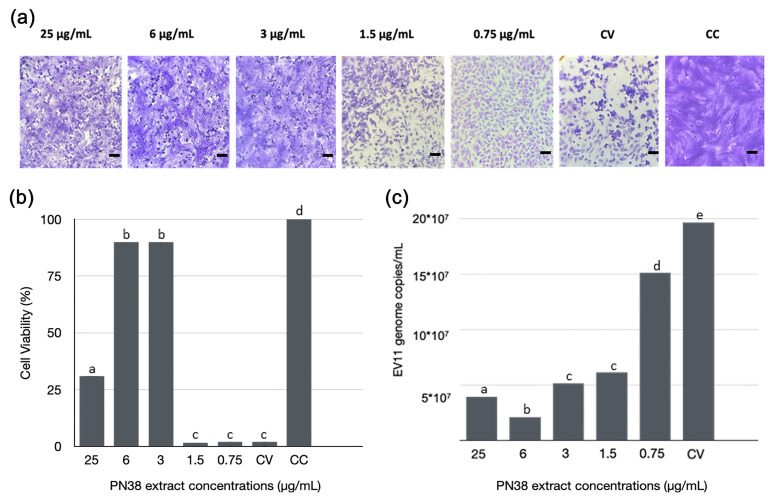
(**a**,**b**) Cytopathic effect inhibition assay. Vero-76 cells infected with E11 (MOI 0.01) treated with different concentrations (25–0.75 µg/mL) of MEAs CHCl_3_ PN38 extract. Control Virus (CV-untreated cell cultures infected with E11) and Control Cells (CC—uninfected and untreated cell cultures) were used as positive and negative controls, respectively. (**a**) Microscopy images of crystal violet stained cells. Scale bar: 100 µm. (**b**) Cell viability of infected and treated Vero-76 cells and relative controls. (**c**) Genome quantification in Vero-76 infected cells, viral RNA detected in the supernatant of Vero-76 cells infected with E11 and treated with PN38 extracts and relative controls (CC and CV) was quantified; the genome copies are reported in scientific notation, the symbol * represents ×. Different letters represent significant differences (*p* < 0.05, Post Hoc test).

The potential antiviral activity of strain PN38 was evaluated by assessing its ability to inhibit viral replication in cell culture. The assay measured the reduction in the cytopathic effect (CPE) induced by Echovirus 11 (E11) in comparison with two controls: a negative control (non-infected cell cultures) and a positive control (infected cell cultures not treated with PN38) [43]. The co-treatment assay with MEAs CHCl_3_ extract (25–0.75 μg/mL) and E11 (MOI of 0.01), showed a strong antiviral effect, with a 90% CPE reduction at 3 and 6 μg/mL, identifying 3 μg/mL as the MIC (Figure 7). This activity was confirmed by the reduction of viral RNA levels in infected Vero-76 supernatants, 86% and 94% at 3 and 6 μg/mL, respectively, compared to the untreated cells (CV) (Figure 7c).

## 4. Discussion

Marine fungi have long been considered an “exotic” group of microorganisms with low species richness and abundance [46]. However, it has become increasingly evident that they represent a substantial proportion of marine microbial diversity and contribute to various key ecological processes in the marine environments (e.g., aquatic carbon pump efficiency and regulation of phytoplankton composition). Despite recent progress in marine mycology, our knowledge of marine fungi remains limited. In this context, research focused on underexplored habitats and substrates can significantly enhance our understanding of these neglected organisms, particularly regarding their distribution, ecology, and contributions to marine ecosystem services. It is worth noting that in recent years, numerous new taxa have been established from marine substrata that had not previously been studied mycologically [32,47].

The seven strains investigated in this study were isolated from the leaves of the seagrass *P. oceanica* (IG108, IG114) and the inner tissues of the jellyfish *P. noctiluca* (PN23, PN28, PN32, PN38, PN39) [3,27]. *P. oceanica* is an endemic seagrass of the Mediterranean Sea; the seagrass meadows rank amongst the most valuable coastal ecosystems on Earth in terms of benefits and services they provide. *P. oceanica* is one of the most studied substrata from marine mycologists [27,48,49,50,51,52,53,54,55,56]. In contrast, *P. noctiluca* mycobiota was only studied by Pasqualetti and co-authors [3]. The isolated strains do not produce reproductive structures on MEAs, utilized for their maintenance. The experiments to promote sexual or asexual reproduction [57,58,59] led only to the development of coelomycetous conidiomata (asexual structures) in PDAs, CMAs and OAs and natural inoculated substrata (barks of *Q. robur*, *P. halepensis*, pine needles of *P. halepensis*, twigs of *T. gallica*, *T. africana* and *Posidonia* leaves) while ascomata were not detected.

A preliminary molecular characterization based on nrITS, nrLSU, and nrSSU allowed us to place the strains IG108, IG114, PN23, PN28, PN32, PN38, PN39 in the family Pleosporaceae [3]. The analysis suggested that the strains were strictly related to the monospecific genus *Tamaricicola* and also indicated that they could represent a new lineage inside the genus [3,27]. To confirm these preliminary observations, a multi-locus phylogenetic analysis, based on molecular markers usually utilized to study the taxonomy of the Pleosporaceae family, was performed [16,17]. The phylogenetic tree (Figure 1), including the genera of the family Pleosporaceae, showed that the strains formed a well-supported clade within the genus *Tamaricicola*, separated from the species *T. muriformis*. Thus, the new species *T. fenicei* was proposed and established. *T. fenicei* is the second species ascribed to the genus *Tamaricicola*. Nevertheless, in the literature some other strains are attributed to the genus *Tamaricicola*: three terrestrial strains isolated from *L. majus* and *L. insigne*, two isolated from avian lung and a marine strain (CHG59) isolated from the gut of the ascidian *C. intestinalis* sampled in the German North Sea [18,19,20].

The nrITS phylogenetic analysis, including all strains attributed to *Tamaricicola,* clearly reveals the presence of three distinct evolutionary lineages: one corresponding to the terrestrial strains of *T. muriformis*, another to the marine strains of *T. fenicei*, and a third comprising the strains isolated from *L. majus* and *L. insigne*. González-Menéndez and collaborators [18] suggested that the *Limonium*-associated strains might represent a novel species within *Tamaricicola*. However, the phylogenetic analysis conducted in this study (Figure 2) indicates that these strains may instead constitute a distinct lineage within the recently established genus *Cnidariophoma* [45], rather than a new lineage within *Tamaricicola*.

Considering the ecology of *T. fenicei*, it is worth noting that strains IG108, IG114, and PN23, PN28, PN32, PN38, and PN39 were isolated from different types of substrates (plant and animal) during two sampling campaigns in the same area (Cala Cupa—Giglio Island) in the Central Tyrrhenian Sea. Thus, it seems reasonable that this species could have a not-specialized habitus [60] even considering the similarity, and probably identity, with the strain *Tamaricicola* sp. CHG59 isolated from *C. intestinalis*. Nevertheless, up to this date the new species seems strictly marine with biontic behavior. The identification of novel taxa significantly contributes to the advancement of knowledge on marine fungi, confirming that the marine ecosystem represents a vast, largely unexplored reservoir of biodiversity and chemo-diversity in particular for its microbial components. It is worth noting that new lineages could be extremely interesting from a blue-biotechnological perspective considering the biological activity of their extract.

The preliminary results of this study revealed the potential in vitro antiviral activity of the PN38 extract against the newly identified E11 variant. Specifically, co-treatment with PN38 extract showed an inhibition of viral replication evaluated by cytopathic effect (CPE) reduction in Vero-76 cells, confirmed by the decrease of viral RNA levels by 86% and 94% at concentrations of 3 and 6 μg/mL, respectively, identifying 3 μg/mL as the MIC.

This value appears to be quite low if compared to similar research, even if carried out on other viruses. In recent publications the 50% of inhibition (EC50: half maximal effective concentration, or concentration of a compound that reduces viral infectivity by 50%) was obtained with similar or much higher concentrations of crude extracts [43,61]. For example, Florio and coworkers [43] considered the range 1.18 to 45 μg/mL of EC50 activity as significant for the selection of fungal strains for further deeper investigations in order to identify new antiviral compounds. These preliminary findings suggest that PN38 extract may contain compounds capable of inhibiting E11 replication in Vero-76 cells, underscoring its potential as a source of secondary metabolites with antiviral properties.

In view of this, the potential in vitro antiviral activity of the PN38 extract against the E11 variant represents a valuable assumption for the future development of new antiviral leads. This work is the first report on antiviral activity of *T. fenicei*; the next step will be the purification and characterization of antiviral compounds and the study of their mechanism of action.

In addition, the ability of *n*-BuOH extract of PN38 (growth in MEAs) to inhibit biofilm formation represents a noteworthy biological activity. The control of infectious diseases associated with biofilms remains a significant challenge, and there is a pressing need for new, effective molecules. The extract from *T. fenicei* showed no activity at the screening concentration against planktonic microbial tested strains; however, it has the ability to tackle the growth as a sessile multistratified community (biofilm) of the relevant nosocomial pathogen *S. aureus*. Biofilm formation is a common virulence factor contributing to invasiveness, ability to evade the host’s defence system, persistence of infection and antibiotic resistance. The preliminary observation of the significant decrease in biofilm formation at low concentration (BIC_50_ equal to 53 µg/mL) of PN38 extract, encourages us to carry out more in-depth studies, for example those concerning interference with regulatory mechanisms of biofilm formation [62]. Although further studies are necessary to identify the specific bioactive compounds of the tested extract and to elucidate their mechanisms of action, the results suggest that the *T. fenicei* PN38 strain is a promising producer of compounds with both antiviral and anti-biofilm activities.

## 5. Conclusions

This study provides a morphological and phylogenetic characterization of seven strains isolated from *P. oceanica* leaves and the inner tissues of *P. noctiluca* collected in the central Tyrrhenian Sea; the mycobiota of this jellyfish has not been previously investigated. The strains represent a new lineage within the genus *Tamaricicola* (family Pleosporaceae) leading to the establishment of the new species *Tamaricicola fenicei*. The identification of new species contributes to the advancement of knowledge about this genus that until now included only a terrestrial species. It is worth noting that all strains attributed to *Tamaricicola fenicei* derived from marine substrata. Finally, the CHCl_3_ extract of *T. fenicei* PN38 exhibits significant antiviral activity against Echovirus 11, while the *n*-BuOH extract markedly reduces biofilm formation by *S. aureus* suggesting that this strain may have high biotechnological potential for drug discovery.

The emergence of drug-resistant pathogens and novel infections has moved toward the sea the search for new pharmaceuticals, and undoubtedly the potential of marine fungi, particularly of novel taxa, in this context is very promising.

## Figures and Tables

**Table 1:** PCR amplification protocols used for the different markers.

Molecular Marker (Primer F/Primer R)	Thermocycler Conditions	References
nrITS (ITS5/ITS4)	94 °C × 2 min, (94 °C × 40 s, 55 °C: 30 s, 72 °C: 45 s) × 35 cycles; 72 °C: 10 min	[33]
nrLSU (LR0R/LR7)	95 °C: 10 min, (95 °C: 60 s, 50 °C: 30 s, 72 °C: 90 s) × 40 cycles; 72 °C: 10 min	[34]
nrSSU (NS1/NS4)	95 °C: 10 min, (95 ◦C: 60 s, 50 °C: 30 s, 72 °C: 90 s) × 40 cycles; 72 °C: 10 min	[33]
*tef-1α* (EF1-983F/EF1-2218R)	95 °C: 10 min, (95 °C: 30 s, 55 °C: 30 s, 72 °C: 60 s) × 40 cycles; 72 °C: 10 min	[35]
*rpb2* (fRPB2-5F/fRPB2-7CR)	94 °C: 5 min, (94 °C: 45 s, 60 °C: 45 s, 72 °C: 120 s) × 5 cycles; (94 °C: 45 s, 58 °C: 45 s, 72 °C: 120 s) × 5 cycles; (94 °C: 45 s, 54 °C: 45 s, 72 °C: 120 s) × 30 cycles; 72 °C: 8 min	[36]

nrITS = nuclear ribosomal Internal Transcribed Spacer, nrLSU = nuclear ribosomal Large SubUnit; nrSSU = nuclear ribosomal Small SubUnit; *tef-1α* = translation elongation factor; *rpb2* = RNA polymerase II subunit.

**Table 2:** Taxa used for the phylogenetic analyses and GenBank accession number. Newly generated sequences are indicated in bold.

Species	Strain	GenBank Accesion Number				
		nrITS	nrLSU	nrSSU	*rpb*2	*tef*-1α
*Alternaria alternata*	MFLUCC 14-1184	KP334711	KP334701	KP334721	KP334737	KP334735
*A. alternata*	MFLUCC 14-0756	KP334712	KP334702	KP334722	KP334738	KP334736
*Alternaria solani*	CBS 116651	KC584217	KC584306	KC584562	KC584430	KC584688
*Alternaria tenuissima*	MFLUCC140441	KU752186	KU561876	KU870907	-	KU577440
*Alternaria cruciatus*	CBS 171.63	MH858254	MH869856	-	ON703247	ON542234
*Alternaria cruciatus*	CBS 536.92	ON773141	ON773155	-	ON703248	ON542235
*Bipolaris cynodontis*	ICMP 6128	JX256412	JX256380	-	-	JX266581
*Bipolaris maydis*	CBS 136.29	MH855024	MH866491	-	HF934828	-
*Bipolaris melinidis*	BRIP 12898	JN192382	JN600994	-	-	KM093771
*Clathrospora elynae*	CBS 196.54	MH857290	KC584371	KC584629	KC584496	-
*Clathrospora elynae*	CBS 161.51	KC584370	KC584628	KC584495	-	-
*Cnidariophoma eilatica*	CPC 44117	OQ628480	OQ629062	-	OQ627943	-
*Comoclathris incompta*	CBS 467.76	KY940770	GU238087	GU238220	KC584504	-
*Comoclathris sedi*	MFLUCC13-0817	KP334715	KP334705	KP334725	-	-
*Comoclathris spartii*	MFLUCC13-0214	KM577159	KM577160	KM577161	-	-
*Curvularia heteropogonis*	CBS 284.91	MH862253	LT631396	-	HF934821	-
*Curvularia lunata*	CBS 730.96	MG722981	LT631416	-	HF934813	-
*Curvularia ravenelii*	BRIP 13165	JN192386	JN601001	-	-	JN601024
*Decorospora gaudefroyi*	CBS 250.60	MH857974	MH869526	-	-	-
*Decorospora gaudefroyi*	CBS 332.63	MH858305	MH869915	-	-	-
*Dichotomophthora lutea*	CBS 145.57	MH857676	NG069497	-	LT990634	-
*Dichotomophthora portulacae*	CBS 174.35	NR158421	MH867137	-	LT990638	LT990668
*Exserohilum monoceras*	CBS 239.77	LT837474	LT883405	-	LT852506	-
*Exserohilum rostratum*	CBS 128061	KT265240	MH877986	-	LT715752	-
*Exserohilum turcicum*	CBS 387.58	MH857820	LT883412	-	LT852514	-
*Gibbago trianthemae*	NFCCI 1886	HM448998	-	-	-	-
*G.ibbago trianthemae*	GT-VM	KJ825852	-	-	-	-
*Halojulella avicenniae*	BCC 20173	-	GU371822	GU371830	GU371786	GU371815
*Johnalcornia aberrans*	CBS 510.91	MH862272	KM243286	-	LT715737	-
*J.ohnalcornia aberrans*	BRIP 16281	KJ415522	KJ415475	-	-	KJ415473
*Neostemphylium polymorphum*	FMR 17886	OU195609	OU195892	-	OU196009	ON368192
*Neostemphylium polymorphum*	FMR 17889	OU195610	OU195914	-	OU196957	ON368193
*Neostemphylium polymorphum*	FMR 17893	OU195631	OU195915	-	OU197255	ON368194
*Paradendriphyella arinariae*	CBS 181.58	MH857747	KC793338	NG062992	DQ435065	-
*Paradendriphyella salina*	CBS 302.84	MH873443	KC584325	KC584583	KC584450	KC584709
*Paradendriphyella salina*	CBS 142.60	MH857928	MH869472	KF156098	DQ435066	DQ414251
*Porocercospora seminalis*	CPC 21332	HF934941	HF934862	-	HF934843	-
*Porocercospora seminalis*	CPC 21349	HF934945	HF934861	-	HF934845	-
*Pyrenophora dictyoides*	DAOM 75616	JN943654	JN940079	JN940962	JN993617	-
*Pyrenophora dictyoides*	DAOM 63666	JN943653	JN940080	-	-	-
*Pyrenophora phaeocomes*	DAOM 222769	JN943649	JN940093	JN940960	DQ497614	DQ497607
*P. pseudoerythrospila*	CBS 127931	NR164465	NG066344	-	-	-
***Tamaricicola fenicei***	**IG108**	**MG977425**	**MG976984**	**MG976980**	**PX411237**	**PX431678**
***Tamaricicola fenicei***	**IG114**	**-**	**PX091581**	**PX412374**	**-**	**PX431679**
***Tamaricicola fenicei***	**PN23**	**OP793911**	**PX412372**	**PX412375**	**PX431674**	**PX431680**
***Tamaricicola fenicei***	**PN28**	**OP793912**	**PX412373**	**PX412376**	**PX431675**	**PX431681**
***Tamaricicola fenicei***	**PN32**	**OP793995**	**PX412371**	**PX412377**	**PX431676**	**PX431682**
***Tamaricicola fenicei***	**PN38**	**ON807355**	**PX091580**	**PX412378**	**ON887328**	**ON952522**
***Tamaricicola fenicei***	**PN39**	**OP794025**	**PX412370**	**PX412379**	**PX431677**	**PX431683**
*Tamaricicola muriformis*	MFLUCC150488	KU752187	KU561879	KU870909	KU820870	KU577441
*Tamaricicola muriformis*	MFLUCC150489	KU752188	KU729857	KU870910	-	KU600013
*Tamaricicola muriformis*	MFLUCC150490	KU752189	KU729856	KU870911	-	KU600014
*Tamaricicola muriformis*	MFLUCC160488	KU900317	KU900293	KU870908	-	-
*Tamaricicola* sp.	CF-288959	MG065814	-	-	-	-
*Tamaricicola* sp.	CF-288916	MG065815	-	-	-	-
*Tamaricicola* sp.	CF-090279	MG065816	-	-	-	-
*Tamaricicola* sp.	CHG59	MW064152	-	-	-	-
*Tamaricicola* sp.	56405C	PP804371	-	-	-	-
*Tamaricicola* sp.	56406A	PP804313	-	-	-	-
*Scleromyces submersus*	FMR 18289	OU195893	OU195959	-	OU197244	OU196982
*Stemphylium lycopersici*	CNU 070067	JF417683	-	-	JF417698	JX213347
*Stemphylium vesicarium*	CBS 191.86	MH861935	JX681120	-	KC584471	KC584731
*Stemphylium vesicarium*	MFLUCC 140442	KU752185	KU561878	KU870906	-	-
*Typhicola typharum*	CBS 145043	MK442590	MK442530	-	MK442666	MK442696

nrITS = nuclear ribosomal Internal Transcribed Spacer, nrLSU = nuclear ribosomal Large SubUnit; nrSSU = nuclear ribosomal Small SubUnit; *tef-1α* = translation elongation factor; *rpb2* = RNA polymerase II subunit.

## Data Availability

The original contributions presented in this study are included in the article. Further inquiries can be directed to the corresponding author.
